# Inflammation: Gone with Translation

**DOI:** 10.1371/journal.pgen.1004442

**Published:** 2014-06-19

**Authors:** Carola G. Vinuesa, Thomas Preiss

**Affiliations:** 1 Dept of Pathogens and Immunity, John Curtin School of Medical Research, Australian National University, Canberra, Australia; 2 Dept of Genome Biology, John Curtin School of Medical Research, Australian National University, Canberra, Australia; The University of Queensland, Australia

Inflammation (derived from the Latin *inflammo*, which means “I set alight”) is a beneficial response of our immune system that protects us against infection and tissue injury. Like all immune responses, it needs to be tightly controlled: excessive inflammatory responses can lead to both acute diseases, such as septic shock, and chronic diseases, such as rheumatoid arthritis, atherosclerosis, and cancer. Until recently, it was thought that the negative regulatory loops that kick in early to counteract inflammation were mainly a result of changes in mRNA levels either through transcriptional activation of inhibitory proteins or posttranscriptional repression of proinflammatory molecules by RNA-binding proteins (RBP) or microRNAs. A paper by Georg Stoecklin and colleagues at the German Cancer Research Center now challenges this notion by demonstrating that, in the early stages of macrophage activation, translational derepression is a major mechanism that induces feedback inhibitors to dampen inflammation [1].

Although neutrophils are the first cells that localise to sites of tissue injury or infection, it is macrophages that orchestrate the multiple components of the inflammatory response through their ability to sense microbial products such as lipopolysaccharides (LPS), which bind toll-like receptor 4 (TLR4). TLR4 ligation initiates a signalling cascade in macrophages that leads to the production of proinflammatory molecules, of which tumor necrosis factor (TNF) is one of the most important [2]. TLR4 signalling and TNF signalling activate several transcription factors, most prominently NF-κB (nuclear factor κ-light-chain-enhancer of activated B cells), that are important for the elimination of pathogens, irritants, or dead cells. Equally important for the healing of tissues later in the inflammatory response is the production of negative regulators of the NF-κB pathway and other inflammatory pathways, which limit the amount of inflammatory mediators and curtail the response in a timely manner [3].

Over the last few years, a number of studies have investigated global gene activation induced by pathogen-derived stimuli to gain insights into the modes of induction of the inflammatory mediators and regulators. Studies focusing on the early stages of inflammation only assessed changes in mRNA levels [4] and identified the importance of mRNA stability in the temporal control of inflammatory molecules. Parallel studies that specifically interrogated translational changes only looked at later stages of the response and were performed in cells other than macrophages [5], [6]. Of note, these latter studies reported translational inhibition of ribosomal proteins and RNAs encoding chemoattractants and their receptors. The present study by Schott et al. [1] is the first to investigate translational regulation in the early phase of macrophage activation and to identify translational derepression as an important mechanism underpinning negative feedback of the inflammatory response.

**Figure 1:**
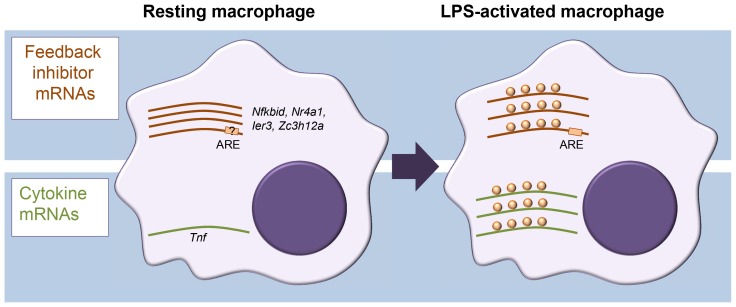
Different modes of mRNA regulation in inflammation. Diagram representing differential control of feedback inhibitor mRNAs and cytokine mRNAs in resting versus activated macrophages. In the resting state, feedback inhibitor mRNAs are being transcribed but translationally repressed; they become derepressed in the early phase of activation. By contrast, most cytokine mRNAs, which are generally unstable, are not expressed in resting macrophages, and their steady-state levels increase quickly upon activation.

In assessing translation, sucrose density gradient centrifugation of cell extracts is often used to separate cellular mRNAs by their association with ribosomes. In combination with microarray analysis, this approach is known as translation state array analysis (TSAA). TSAA measures the proportion of mRNAs in dense polysomal complexes as an indicator of their translational activity [7]. The investigators treated cultures of the murine RAW264.7 macrophage cell line with LPS for 1 h and performed TSAA, in addition to measuring mRNA steady-state levels in parallel by RNA-seq. This combination allowed them to focus on mRNAs with an active change in ribosome load, identifying 59 cases of translational up-regulation and 55 cases of down-regulation, many of which could be validated by quantitative polymerase chain reaction (qPCR) not only in the original cell line but also in primary cultured macrophages. Four cytokine mRNAs scored as up-regulated (Tnf, Cxcl2, Il23a, and Tnfsf9) while, surprisingly, several mRNAs encoding proteins classed as feedback inhibitors of TLR4 signalling were also actively derepressed in their translation (Figure 1). Among the latter were those encoding likely inhibitors of NF-κB activation (IκBδ, IER3, and NR4A1), the p38 mitogen-activated protein kinase (MAPK) pathway inhibitor dual specificity phosphatase 1 (DUSP1), as well as the RBPs ZFP36 and ZC3H12A, known inhibitors of cytokine expression. Most cytokine mRNAs were scarce in resting macrophages and strongly induced. By contrast, feedback inhibitor mRNAs tended to already be abundant and showed relatively modest increases in steady-state level upon activation.

A motif search by Schott et al. [1] identified an overrepresentation of the adenylate-uridylate-rich element (AU-rich element; ARE) within the 3′ untranslated regions (UTRs) of translationally up-regulated mRNAs. AREs are present in the 3′ UTR of many cytokine mRNAs, including the *Tnf* mRNA, and recruit a range of RBPs to regulate mRNA stability and translation [8]. The authors further investigated the example of IER3 mRNA 3′ UTR to show that the ARE element was required for repression in resting cells as well as derepression after LPS induction. The present study makes no further inroads into identifying the molecular players and mechanism involved in this ARE-mediated repression/derepression. One intriguing possibility we see is that it could be driven by a switch to aerobic glycolysis commonly seen in activated immune cells [9] and involve ARE-binding by the glycolysis enzyme glyceraldehyde 3-phosphate dehydrogenase (GAPDH), as reported for interferon γ (IFNγ) mRNA during T cell activation [10]. Binding of metabolic enzymes to RNA has been repeatedly reported and might underpin a broader crosstalk between metabolism and gene regulation and offer novel therapeutic possibilities [11].

The corollary from the studies by Schott et al. [1] is that, upon macrophage activation, expression of several anti-inflammatory inhibitors is driven by translational up-regulation of preexisting mRNAs. That cells choose the relatively resource-costly approach of stockpiling these mRNAs highlights their need to swiftly mount a feedback response to contain the inflammatory response. By contrast, induction of proinflammatory cytokines is predominately achieved by changes in mRNA abundance. This adds a new facet to the multilevel gene regulatory network controlling the inflammatory response. While we have known about the post-transcriptional regulation of cytokine mRNAs for some time, there is now a need to better understand how cells ensure the swift production of feedback inhibitors using translational control.
